# Investigating Methods to Mitigate Whey Protein Derived Mouthdrying

**DOI:** 10.3390/foods10092066

**Published:** 2021-09-01

**Authors:** Victoria Norton, Stella Lignou, Marianthi Faka, Julia Rodriguez-Garcia, Lisa Methven

**Affiliations:** 1 Department of Food and Nutritional Sciences, Harry Nursten Building, University of Reading, Whiteknights, Reading RG6 6DZ, UK; v.l.norton@pgr.reading.ac.uk (V.N.); s.lignou@reading.ac.uk (S.L.); j.rodriguezgarcia@reading.ac.uk (J.R.-G.); 2 Volac International Limited, 50 Fisher’s Lane, Royston SG8 5QX, UK; marianthi.faka@volac.com

**Keywords:** whey protein, mouthdrying, sensory, mitigating strategies

## Abstract

Mouthdrying is commonly associated with whey protein fortified products. Therefore, mitigating strategies could be key to reducing mouthdrying and maximising the benefits from such products. Currently, few studies have successfully mitigated whey protein derived mouthdrying and this paper aims to investigate different strategies to reduce mouthdrying effects. Accordingly, a series of experiments were carried out with a trained sensory panel (*n* = 11). Two different whey protein food matrices were tested: (a) whey protein beverages (WPB) varying in lactose (0.05–12.4% *w*/*v*) and fat (0.9–7.2% *w*/*v*) levels and (b) whey protein fortified snacks: cupcakes with differing whey protein concentrate (WPC) powders (standard and heat-stable) and scones with varying fat content (with and without cream topping). Overall results suggested the tested strategies had limited significant effects on whey protein derived mouthdrying. Increasing lactose (9.4% *w*/*v*) in WPBs and fat levels (via cream topping) on scones significantly suppressed mouthdrying. However, all other tested strategies (increasing fat in WPBs and heat-stable WPC in cupcakes) had no significant effect on suppressing perceived mouthdrying. This work demonstrates the challenges with mitigating whey protein derived mouthdrying; however, cross-modal taste suppression and increasing lubrication warrant further investigation.

## 1. Introduction

Whey protein can be described as a value-added ingredient due to its well-cited nutritional and health benefits [1,2]. Accordingly, whey protein is often fortified into different food matrices to enhance protein intake; such applications typically include the older consumer (to help prevent malnutrition and sarcopenia) or the sport, health and lifestyle consumer (to enhance performance or health) [3]. However, regardless of the application, the sensory profile of such products is key to consumer acceptance and subsequent consumption. As alluded to in our recent review, negative sensory attributes are associated with whey protein fortified products, more specifically, mouthdrying [4].

Whey protein derived mouthdrying can be described as the drying sensation in the mouth during or post consumption [4,5,6,7]. In addition, mouthdrying and/or dry texture is present in both liquid and solid models fortified with whey protein, such as cakes, beverages, biscuits, muffins and rye breads [5,6,7,8,9,10]. To date the causes of such mouthdrying are inconclusive; however, adhesion of protein to the oral cavity (mucoadhesion) has been found to correlate with mouthdrying perception in whey protein beverages (WPB) [7]. This is highly relevant to older consumers as mouthdrying sensitivity and mucoadhesion are both considered to increase with age [5,11]. Therefore, strategies to mitigate mouthdrying are key to promoting consumer compliance and acceptance.

Despite mouthdrying being present in a range of whey protein fortified products, there are few studies which have successfully mitigated whey protein derived mouthdrying. For example, Withers et al. [12] focused on three strategies, namely increasing sweetness (3% *wt*/*wt* sucrose), viscosity (1.8% *wt*/*wt* starch thickener) and fat (2% *wt*/*wt* sunflower oil and milk fat) based on previous astringency work. It was suggested that sweetness could suppress mouthdrying, viscosity could reduce interactions within the oral cavity and fat could improve lubrication; therefore, subsequently reducing mouthdrying [12]. However, the results using a trained sensory panel suggested such strategies at the levels tested had a limited effect on perceived mouthdrying [12]. In brief, increasing sweetness and fat significantly increased mouthdrying to a small extent, whilst viscosity had a small significant effect in suppressing mouthdrying in a protein-fortified milk matrix, but there was no significant reduction in the complete oral nutritional supplement (ONS) [12]. Therefore, concluding more research would be beneficial to understand better strategies to effectively mitigate mouthdrying.

Various other studies have shown that sweetness could have a role in suppressing dairy or plant based mouthdrying. Methven et al. [13] demonstrated in ONS (standard ONS vs sweetness suppressed ONS) that increased sweetness correlated with reduced mouthdrying. This finding was supported by two additional studies: (1) soymilks with increased sucrose reduced astringency [14] and (2) adding sucrose and vanilla flavouring suppressed mouthdrying in WPBs [7]. However, these studies were limited as they added a set amount of sugar to increase sweetness, rather than a progression to understand at what point sweetness could suppress mouthdrying. 

Withers et al. [12] used sunflower oil and milk fat to fortify dairy beverages, yet they found that these fats at the levels used (2% *wt*/*wt*) were unable to suppress mouthdrying in the liquid beverage. However, other fat sources or levels may have an effect. For example, where cream was added to skimmed milk, varying in fat content (0.2–5% *wt*/*wt*), the higher fat levels (2 or 5% *wt*/*wt*) were found to reduce perceived astringency [15]. Therefore, adding fat to WPBs could influence mouthdrying, but this is yet to be fully investigated. In addition, Engelen, Fontijn-Tekamp and van der Bilt [16] noted that increasing fat levels (by adding a topping such as butter) to solid food models could reduce the number of chews, via increased lubrication. Similarly, utilising toppings (firm cheese, cheese spread and mayonnaise) decreased dryness and firmness in bread and crackers [17]. Accordingly, adding toppings to whey protein fortified solid foods could be a potential strategy to suppress whey protein derived mouthdrying.

Previous research suggests that heat treatment of WPBs (unheated vs heated for 20-min) alters the mouthfeel attributes (increasing body, chalky, mouthdrying, mouthcoating and furring) [8]. In addition, differences in processing can impact the functional properties of whey proteins [18]. This suggests whey protein powders (such as whey protein concentrate, WPC), which have undergone an additional heat treatment process during manufacturing, could influence subsequent texture perception of fortified products [19]. For example, a heat-stable WPC could lead to a creamier mouthfeel in a product compared with a standard WPC, potentially by increasing flow and reducing friction [19,20,21]. Accordingly, this warrants investigation into its subsequent effects on perceived mouthdrying within a solid food matrix.

Currently, few studies have effectively suppressed whey protein derived mouthdrying in either a liquid or solid model and a more fundamental investigation is needed. Mitigating mouthdrying could create more acceptable products and promote product consumption. This paper hypotheses that mitigating strategies will reduce mouthdrying as follows: (a) lactose will suppress mouthdrying via cross-modal suppression; (b) increasing lubrication via fat will suppress mouthdrying; and (c) heat-stable WPC in cupcakes will reduce mouthdrying. This paper tests whether these strategies can reduce perceived mouthdrying in two different whey protein food matrices (liquid and solid model), using a sensory trained panel.

## 2. Materials and Methods

### 2.1. Overview of Experiments

**Figure 1:**
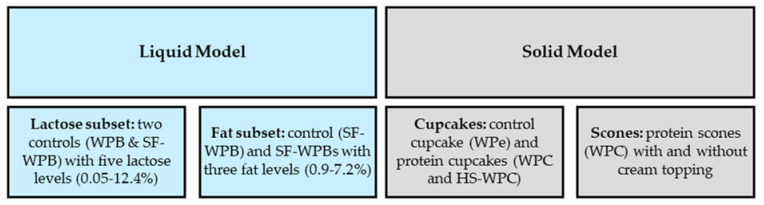
Overview of experiments (WPB: whey protein beverages; SF-WPB: sugar-free whey protein beverage; WPe: whey permeate; WPC: whey protein concentrate; HS-WPC: heat-stable whey protein concentrate). Brackets after each sample name denote specific lactose or fat content expressed as % *w*/*v*.

A series of experiments (as outlined in Figure 1) were conducted using a trained screened experienced sensory panel (*n* = 11; 10 female and 1 male). The experiments were not subjected to a specific ethical review nor additional consent, as the trained sensory panel were tasting products made from standard commercial food ingredients. However, it should be noted that all panellists had consented to evaluate different food and beverage products as part of their employment contract.

### 2.2. Materials

Volac (Volac International Ltd., Royston, UK) provided five different whey derived powders: (1) whey protein concentrate instantised with sunflower lecithin (WPC, Volactive^®^ UltraWhey Instant 80; 81% protein); (2) sugar-free whey protein concentrate instantised with sunflower lecithin (SF-WPC, Volactive^®^ UltraWhey Sugar Free WPC Instant; 86% protein); (3) heat-stable whey protein concentrate (HS-WPC, Volactive^®^ UltraWhey Velicious^TM^; 70% protein); and (4) whey permeate (WPe, Volactose^®^ Taw Whey Permeate; 89% lactose) and (5) lactose (Volactose^®^ Edible Lactose; 99% lactose). Maltodextrin and xanthan gum-based thickener (Nestle Resource Thicken Up Clear) was obtained from NutriDrinks (London, UK). Soya lecithin (Louis Francois, Lecithine De Soja En Poudre I.P.-E322) was acquired from Sous Chef (London, UK). Baking ingredients, double cream (British Double Fresh Cream, UK) and clotted cream (Rodda’s Clotted Cream, Cornwall) were all purchased from Sainsburys (Reading, UK).

### 2.3. Whey Protein Liquid Models

#### 2.3.1. Lactose Subset

Two control beverages were tested: (a) whey protein beverage (WPB, 10% *w*/*v* WPC powder in deionised water) and (b) sugar-free whey protein beverage (SF-WPB, 10% *w*/*v* SF-WPC powder in deionised water). SF-WPB was fortified with lactose at five different levels to represent a range from 0.4 to 12.4% *w*/*v*, based on a ×3.0 progression. The rationale for the lactose levels was that 0.4% *w*/*v* matches the control WPB lactose levels, 3.4% *w*/*v* is considered just below the lactose relative sweetness detection threshold [22] and 12.4% *w*/*v* provides a similar relative sweetness level (~2% *w*/*v* sucrose) to our previous work [7].

#### 2.3.2. Fat Subset

The control beverage was a sugar-free whey protein beverage (SF-WPB, 10% *w*/*v* SF-WPC powder in deionised water). Double cream was added to SF-WPB at three different levels (1.8%, 3.6% and 7.2% *w*/*v*) to represent the mid-range fat levels found in ONS. A hydrocolloid (maltodextrin and xanthan gum-based thickener) was added (0.03–0.1% *w*/*v*) to minimise differences in viscosity between fat levels, without influencing flavour or mouthfeel attributes. Lecithin (0.1% *w*/*v*) was also added to ensure a stable dispersion of the fat phase in the beverage. 

All beverages are summarised in Table 1. In both subsets, the preparation method utilised is as described in previous work [5,7], where all beverages were prepared and stirred (Stuart^TM^ SM5 Bibby Fascia, Cole-Parmer, Staffordshire, UK) for 90-min at room temperature (19.3 ± 0.5 °C), hydrated overnight (4 °C) and assessed or consumed at room temperature the following day.

### 2.4. Whey Protein Solid Models

All solid model formulations and nutritional compositions (Nutritics v5.64, Dublin, Ireland) are outlined in Table 2.

#### 2.4.1. Cupcakes

Three different lemon cupcakes were developed based on our previous work [6]. The control cupcake was fortified with whey permeate and the protein cupcakes were fortified with (a) whey protein concentrate (WPC cupcake) and (b) heat-stable whey protein concentrate (HS-WPC cupcake) to understand the influence of processing differences on subsequent perception. The recipes were prepared as previously described [6]. In summary, an all-in-one method was utilised (low speed 5 to 8-min, Kenwood Titanium Major KMM020, Hampshire, UK) until well-mixed and the batter (38.2 g) was individually weighed into paper cases (80 mm × 62.5 mm). Cupcakes were baked at 170 °C for 20-min (in a pre-heated Altas Salva Oven, London, UK). Cupcakes were individually packaged in heat-sealed polypropylene pouches, frozen at 18 °C until time of consumption and a sample (150 g) from each batch was sent for microbiological testing (SYNLAB, Northumberland, UK).

#### 2.4.2. Scones

##### Scones Sensory Profiling

Cupcakes were already considered high in fat content (23–25% *w*/*v*); hence, scones were formulated to investigate the effect of fat on mouthdrying perception. Two different scones were tested: (a) control scone fortified with whey permeate and (b) protein scone fortified with whey protein concentrate. In summary, self-raising flour, sugar, whey powders and butter were mixed until resembling fine breadcrumbs (low speed, 5 to 10-min). Eggs and milk were added and mixed (low speed, 2-min). Dough pieces were rolled (sheeted, 1 cm thickness), cut (using 4.5 cm cutter) and weighed (32.5 g). All tops of scones were brushed with eggs and milk mixture and baked at 200 °C for 12-min. Scones were baked and consumed fresh (within 4-h) for full sensory profiling. The rationale for baking scones fresh related particularly to the control scone being adversely affected by freezing due to starch retrogradation [23] and subsequent staling.

##### Scones with and without Topping

Sensory profiling results (Table S2) demonstrated the key differences between the control and protein scone were mainly related to mouthfeel. Thus, in order to evaluate the effect of fat on mouthfeel perception, only the protein scone was assessed with and without cream topping (8 g of clotted cream providing 5 g of fat). Scones were individually packaged (heat-sealed polypropylene pouches), frozen (−18 °C) until time of consumption and a sample (150 g) from each batch was sent for microbiological testing.

#### 2.4.3. Physical Properties of Cupcakes and Scones

The physical properties of the cupcakes and scones were analysed in triplicate from three different batches (*n* = 9). In brief, the following analysis was carried out based on our previous work [6]: (a) moisture content (%) (moisture analyser, Sartorius MA37, Germany); (b) water activity (a_w_) (Hydrolab C1, UK); (c) crumb colour was measured (colorimeter, Chroma Meter CR-400, Japan) and the results were expressed in accordance with the CIELAB system (illuminant C and 10° viewing angle) where L* (lightness) was recorded and the a* (red-green) and b* (yellow-blue) colour coordinates were converted to the hue angle (arctan (b*/a*)) [24]; (d) height (mm) (digital calipers, Whitworth Tool Inc., USA) and (e) texture profile analysis (TPA) using a double compression test (cylindrical probe, P/75; 15 mm slice) on a texture analyser (XTPlus, Stable Micro System, Godalming, UK).

### 2.5. Sensory Profile

The trained sensory panel (with extensive experience of profiling whey protein fortified products) used quantitative descriptive analysis (QDA^TM^) [25] (in accordance with ISO 8586:2012 and 11132:2012) to determine the sensory profile [26,27,28]. All experiments were carried out at each panellist’s home due to COVID-19 restrictions, whilst adhering to COVID-19 guidelines at the time (January to April 2021) with suitable risk assessments. All sessions were conducted on Microsoft Teams (Version 1.3.00.28778, Washington, DC, USA); scoring was completed individually using Compusense Cloud Software (Version 21.0.7713.26683, Compusense, Guelph, ON, Canada). All samples were prepared at the University of Reading and provided to panellists each morning; testing was completed individually on an iPad (Apple, London, UK) in a quiet and aroma free location. All scoring was conducted in duplicate in separate sessions and on visual analogue scales (VAS; 0–100) with products (coded with a random three-digit number) consumed in a sequential balanced order with randomly allocated sample sets. In all experiments the panellists developed a consensus vocabulary (Table 3) adapted from our previous work [6,7] with modifications for each experiment are summarised in Table 4.

### 2.6. Statistical Analysis

In all experiments, QDA data was analysed using SenPAQ (version 6.3, Qi Statistics, UK) by analysis of variance (ANOVA; rationale as outlined in our previous work [6,7]). The main effects (product and panellist) were tested against the product by panellist interaction (with product and panellists as fixed and random effects respectively). Post hoc analysis (if ANOVA denoted significant value) was carried out using either Fishers least significant difference (LSD) (less than 5 samples) or Tukey-Kramer honestly significant difference (HSD) (5 or more samples) to determine multiple comparisons [29]. 

XLSTAT (version 2020.1.3, Addinsoft, New York, NY, USA) was used to analyse cupcake and scone physical properties data; specific statistical tests were based on distribution of data (normally distributed data as defined by normality of residuals *p* > 0.05) and number of samples: (a) cupcakes via ANOVA (normally distributed data with multiple pairwise comparisons carried out using Fishers LSD) and Kruskal-Wallis test (non-normally distributed data) and (b) scones were analysed using *t*-tests (normally distributed data) and Mann-Whitney test (non-normally distributed data). In all experiments sample significance was defined as *p* < 0.05.

## 3. Results

### 3.1. Whey Protein Beverages with Lactose

Fortifying WPBs with lactose resulted in nine out of 18 attributes being significantly different as demonstrated in Table 5. In brief, to varying extents, increasing lactose significantly reduces sourness, whey isolate, powdery, mouthdrying and metallic notes, as well as significantly increasing sweetness, cooked milk, aftertaste strength and sweet aftertaste. The sensory profile also demonstrated minimal differences between the two controls (WPB and SF-WPB). However, it should be noted that lactose had only a small effect on significantly suppressing mouthdrying, and this was only significant at 9.4% lactose which correlated with high sweetness intensity.

### 3.2. Whey Protein Beverages with Fat

**Figure 2:**
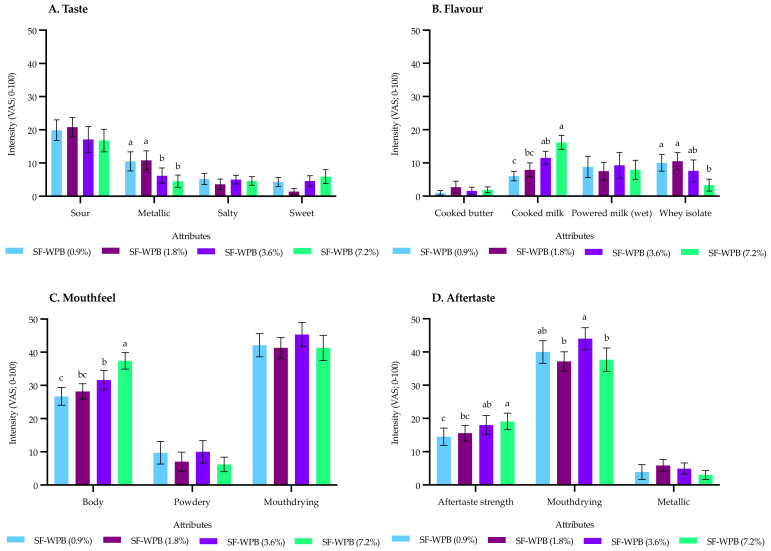
Influence of fat content on the sensory profile of whey protein liquid models (SF-WPB: sugar-free whey protein beverage). Data represents means of two replicates ± standard error from trained sensory panel (*n* = 11) measured on visual analogue scales (VAS; 0–100). Differing small letters represent sample significance from multiple comparisons and brackets after sample name denote specific fat content expressed as % *w*/*v*. All attributes are fully defined in Table 3.

The sensory profile of WPBs, varying in fat, resulted in six significant differences (from 14 attributes) demonstrating fat significantly reduced metallic taste and whey isolate flavour, whilst significantly increasing cooked milk flavour, body, aftertaste strength and mouthdrying aftertaste (Figure 2). In summary, increasing fat (via double cream) had no significant effect on mouthdrying during consumption; however, post consumption (aftertaste) mouthdrying was significant but did not follow a consistent trend with increasing fat levels.

### 3.3. Cupcakes

There were 15 significant differences reported from 37 attributes, as outlined in Table 6. In summary, protein fortification (WPC and HS-WPC cupcake) resulted in significantly increased firmness of bite, chewiness and mouthdrying, whilst significantly reducing moist sponge and rate of breakdown and clearance compared with the control cupcake. Overall, there were minimal differences in the sensory profile between the two protein versions (WPC and HS-WPC cupcakes) with mouthdrying reported to the same extent between the two protein versions. 

The physical properties of cupcakes are summarised in Figure S1, where the heat stable WPC had a greater effect on the physical properties. For example, HS-WPC cupcakes had significantly lower hardness, cohesiveness, chewiness and had a more yellow crumb colour (higher b* and lower hue angle) compared with WPC cupcakes.

### 3.4. Scones

Sensory profiling demonstrated four significant differences from 32 attributes between the control and protein scones, as described in Table S2. Scones fortified with whey protein (WPC) were significantly more savoury/cheesey aroma and mouthdrying, as well as having a significantly less moist appearance and moist dough mouthfeel compared with the control scone.

Whey protein fortification significantly altered the physical properties of the scones, where the protein scone was significantly harder and chewier (Figure S2).

**Figure 3:**
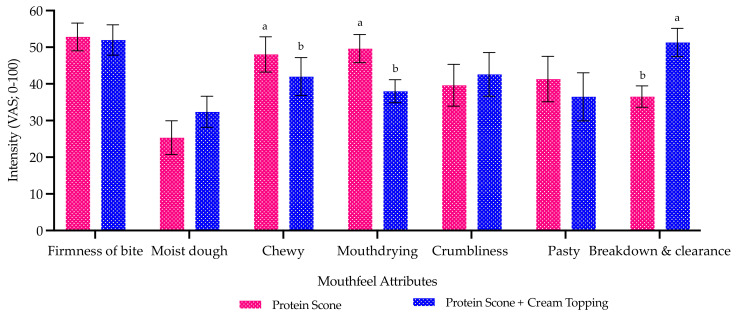
Mean mouthfeel attribute ratings of scones with and without cream topping. Data represents means of two replicates ± standard error from trained sensory panel (*n* = 8) measured on visual analogue scales (VAS; 0–100). Differing small letters represent sample significance from multiple comparisons. All attributes are fully defined in Table 3.

Key mouthfeel attributes (*n* = 7) were assessed for the protein scone with and without the cream topping. This demonstrated that fat (via clotted cream) significantly reduced mouthdrying and chewiness, as well as significantly increasing rate of breakdown and clearance (Figure 3). This concludes that increasing fat levels in scones can significantly suppress mouthdrying.

## 4. Discussion

### 4.1. Whey Protein Beverages with Lactose

SF-WPBs were fortified with lactose at a spectrum of different sweetness levels. However, results suggest lactose was only able to significantly suppress mouthdrying at one of the higher lactose levels (9.4% *w*/*v*) and only to a minor extent. These results imply that a substantial amount of lactose is necessary to reduce mouthdrying and a plateau is reached beyond which further addition has no effect (i.e., at 12.4% *w*/*v* lactose the SF-WPB was not significantly sweeter and mouthdrying was not further reduced). This indicates a cross-modal effect related to the increase in sweetness. Sweetness suppressing mouthdrying is supported by previous work in this area [7,13,14]. Conversely, one study did not find mouthdrying to be reduced by increasing sweetness, this could relate to the beverage models utilised being more complex and involving multiple ingredients (milk protein concentrate, whey protein concentrate and skim milk) or the sensory method employed (sequential profiling) [12]. The proposed mechanism for sweetness suppressing mouthdrying is via a cross-modal cognitive effect rather than a physical change, as a sweetened WPB still adheres to the oral cavity [7]. In addition, the sensory profile results highlighted minimal differences between the two controls: (a) WPB (0.4% *w*/*v* lactose) and (b) SF-WPB (0.05% *w*/*v* lactose). There were no significant differences in sweetness and only slight differences in mouthdrying and aftertaste strength; this could have useful applications for the sport, health and lifestyle consumers interested in products with minimal sugar content. 

### 4.2. Whey Protein Beverages with Fat

Fat provides oral lubrication and alters roughness, friction and creaminess [30], hence adding fat could help to suppress whey protein derived mouthdrying. Accordingly, SF-WPBs were fortified with three different levels of fat (via double cream) and this had no significant effect on mouthdrying during consumption. However, increasing fat content had significant, but mixed effects, post consumption (aftertaste) on mouthdrying: (a) from 1.8% to 3.6% *w*/*v* fat, mouthdrying increased and (b) from 3.6% to 7.2% *w*/*v* fat, mouthdrying reduced. Furthermore, none of the SF-WPBs with added fat were significantly different in mouthdrying aftertaste compared with the control SF-WPB (0.9% *w*/*v* fat). Similarly, Withers et al. [12] also demonstrated that increasing fat (sunflower oil and milk fat at 2% *wt*/*wt*) could result in a significant, but minimal, increase in mouthdrying. However, a previous study which fortified skimmed milk with cream found that the higher fat levels (2 or 5% *wt*/*wt*) correlated with reduced astringency [15]. This suggests that the model beverage could be relevant where the different mechanisms associated with mouthdrying and astringency are potentially different [7] leading to variations in results. It is also noteworthy that fat was able to mask other negative sensory attributes (such as whey isolate and metallic notes), which could also have a positive effect on consumer acceptance. Therefore, altering the fat levels within products could be an alternative approach to improving mouthfeel of WPBs. 

### 4.3. Cupcakes

Differing WPCs (standard and heat-stable) had a minimal effect on the sensory profile, where the perception of the two protein fortified versions was very similar in contrast with the control cupcake. Interestingly, there were significant differences in physical properties resulting in the heat-stable cupcake (HS-WPC) having lower hardness, chewiness and cohesiveness compared with the WPC cupcake. This resulted in a potentially more favourable texture compared with the WPC cupcake; however, these differences had limited effect on the sensory profile. Cake crumb is formed by a two gel-forming system: starch swelling and gelatinisation, and a protein network denaturation and coagulation, both contributing to cake texture (firmness and cohesiveness) [31,32,33]. Hence, it is hypothesised that the HS-WPC powder could have influenced the formation of the starch-filled protein network as a result of the HS-WPC protein particles aggregating with exposed thiol groups, as well as interactions with other sulfhydryl groups from the egg or gluten. This potentially disrupted the network formation during coagulation and resulted in a weaker crumb structure. In addition, the sensory profile and physical properties demonstrated slight colour differences between the cupcakes. The HS-WPC cupcakes generally supported a colour profile more similar to the control cupcakes (i.e., more yellow colour) compared with the WPC cupcakes, as noted particularly by the sensory panel results. It was expected that the additional processes, which result in a heat-stable WPC powder, would impact positively the final product, leading to a creamier and smoother mouthfeel, potentially resulting from improved lubrication and/or reduced adhesion to the oral cavity [5,7,19,34,35]. It is possible that the trained panellists found minimal differences in the sensory profile between the two protein versions due to the cupcake model being relatively high in fat; therefore, any difference in processing or heat treatment of the whey protein, could have a greater effect in other foods models. Previous research, using heat-treated whey protein in liquid and semi-solid models, has demonstrated a positive effect on product sensory profile [35]. Liu et al. noted heat-treated whey protein can result in rough and dry perception if particles sizes are above the detection threshold [35]. Therefore, future studies should consider not only the processing and heat stability of the whey protein powder, but also the particle size.

### 4.4. Scones

As expected, fortifying scones with whey protein (WPC) altered the sensory profile, demonstrating key sensorial issues, namely mouthdrying, supporting previous work in this area [6]. However, this present work hypothesised that the effect of fat could be greater in a solid model (such as scones) than in a liquid model. Engelen et al. [16] noted that hard and dry products typically need more chewing and time in the mouth prior to swallowing. Furthermore, adding butter to cake and toast significantly decreased number of chews, presumably from increased lubrication [16]. In addition, fat is suggested to provide flavour, taste and mouthfeel [21]. Our work builds on the Engelen et al. [16] findings by demonstrating that using a high fat topping can alter the mouthfeel attributes by reducing chewiness and mouthdrying, as well as increasing rate of breakdown and clearance. van Eck et al. [17] also proved that toppings (such as firm cheese, cheese spread and mayonnaise) can reduce dryness and firmness and increase flavour perception of bread and crackers. It was suggested that this is due to saliva aiding bolus formation, whilst the nature of the topping and the product characteristics also influence the extent of change in perception [17]. More specifically in whey protein models incorporating ingredients such as butter into cream cheese improved flavour and liking [10]. Furthermore, this highlights that the use of toppings can make foods more acceptable and reduce negative mouthfeel attributes; accordingly, could be a viable route for improving the protein intake of older adults. 

## 5. Conclusions

This paper demonstrated, despite using four different mitigating strategies in two different whey protein food models, that these strategies had limited effect on suppressing whey protein derived mouthdrying. Fortifying WPBs with lactose significantly reduced mouthdrying to a small extent; however, this correlated with increased sweetness highlighting cross-modality, rather than physical modification, as the probable mechanism. Increasing fat levels in whey protein fortified scones (via clotted cream) significantly reduced mouthdrying. However, increasing fat levels in WPBs did not significantly reduce mouthdrying. Hence, these results suggest increasing lubrication could be more relevant in a solid model compared with the liquid model. Heat-stable WPC in cupcakes had no significant effect on reducing perceived mouthdrying but lead to some improvements in the physical properties compared with WPC cupcake. This work highlights the challenges to mitigating mouthdrying; however, there is a clear need to explore methods of improving lubrication in the mouth. Developing our understanding of the proposed causes of whey protein derived mouthdrying remains key so that fortified products can be reformulated to improve the sensory profile and subsequently mitigate mouthdrying. This has relevance for the growing whey protein fortified products market for both older adults and the sport, health and lifestyle consumers.

## Figures and Tables

**Table 1:** Overview of whey protein liquid model lactose and fat subset (WPB: whey protein beverage; SF-WPB: sugar-free whey protein beverage).

	Lactose Subset								Fat Subset			
	Controls		SF-WPBs Varying in Added Lactose						Control	SF-WPBs Varying in Added Fat		
	WPB (0.4%)	SF-WPB (0.05%)	SF-WPB (0.4%)	SF-WPB (3.4%)	SF-WPB (6.4%)	SF-WPB (9.4%)	SF-WPB (12.4%)		SF-WPB (0.9%)	SF-WPB (1.8%)	SF-WPB (3.6%)	SF-WPB (7.2%)
**Formulations**												
Deionised water (mL)	90	90	90	87	84	81	78		90	88	84	77
WPC (g)	10	-	-	-	-	-	-		-	-	-	-
SF-WPC (g)	-	10	10	10	10	10	10		10	10	10	10
Lactose (g)	-	-	0.4	3.4	6.4	9.4	12.4		-	-	-	-
Double cream (g)	-	-	-	-	-	-	-		-	1.8	5.6	13.2
Lecithin (g)	-	-	-	-	-	-	-		-	0.1	0.1	0.1
Hydrocolloid (g)	-	-	-	-	-	-	-		0.1	0.05	0.03	-
**Composition**												
Energy (kcal)	40.1	41.6	41.8	43.0	44.1	45.3	46.5		41.6	49.5	66.1	99.3
Fat (g)	0.8	0.95	0.95	0.95	0.95	0.95	0.95		0.95	1.8	3.6	7.2
of which saturates (g)	0.3	0.4	0.4	0.4	0.4	0.4	0.4		0.35	0.9	2.0	4.3
Carbohydrate (g)	0.4	0.05	0.4	3.4	6.4	9.4	12.4		0.05	0.08	0.1	0.2
of which sugars (g)	0.4	0.05	0.4	3.4	6.4	9.4	12.4		0.05	0.08	0.1	0.2
Protein (g)	8.2	8.6	8.6	8.6	8.6	8.6	8.6		8.6	8.6	8.7	8.8

Composition data is obtained from ingredients technical sheets and dash (-) represents not applicable. Brackets after each sample name denote specific lactose or fat content expressed as % *w*/*v*. Acronyms: whey protein concentrate (WPC); sugar-free whey protein concentrate (SF-WPC). Viscosity of WPBs were measured to ensure similarity within subsets and summarised in Table S1.

**Table 2:** Overview of whey protein solid model cupcakes and scones (WPC: whey protein concentrate; HS-WPC: heat stable whey protein concentrate) per 100 g.

	Cupcakes				Scones		
	Control	WPC	HS-WPC		Control	Protein	Protein + Cream Topping
**Formulations**							
Unsalted butter (g)	23.0	23.0	22.7		10.2	10.2	10.2
Self-raising flour (g)	23.0	23.0	22.7		46.0	46.0	46.0
Caster sugar (g)	23.0	23.0	22.7		5.1	5.1	5.1
Milk (whole) (g)	5.0	5.0	4.9		20.4	20.4	20.4
Eggs (free-range) (g)	18.6	18.6	18.4		10.2	10.2	10.2
Lemon zest (g)	0.76	0.76	0.75		-	-	-
Whey permeate (g)	6.64	-	-		8.0	-	-
WPC (g)	-	6.64	-		-	8.0	8.0
HS-WPC (g)	-	-	7.7		-	-	-
Clotted cream (g)	-	-	-		-	-	26.7
**Composition**							
Energy (kcal)	442	445	448		350	353	509
Fat (g)	23.0	24.0	25.0		12.0	13.0	30.0
of which saturates (g)	14.0	14.0	14.0		6.9	7.2	17.8
Carbohydrate (g)	51.0	45.0	44.0		52.0	44.0	44.6
of which sugars (g)	26.0	26.0	26.0		15.0	7.5	8.1
Fibre (g)	1.1	1.1	1.1		2.0	2.0	2.0
Protein (g)	6.0	12.0	12.0		7.6	15.0	15.4
Salt (g)	0.2	0.2	0.2		0.5	0.5	0.5

Composition data is obtained from Nutritics software and dash (-) represents not applicable. Acronyms: whey protein concentrate (WPC); heat-stable whey protein concentrate (HS-WPC).

**Table 3:** Summary of quantitative descriptive analysis (QDA) attributes with reference and/or description for all experiments (WPBs with lactose ^1^, WPBs with fat ^2^, cupcakes ^3^, scones ^4^ and scones with and without cream topping ^5^).

Modality	Attribute	Reference and/or Description
Appearance	Moist appearance ^3,4^	Slightly or moderately wet to touch
	Dense appearance of sponge/dough ^3,4^	Compact in structure
	Appearance of large holes in sponge ^3,4^	Holes within crumb/dough structure (none to lots)
	Yellow colour of crumb/dough (inside) ^3,4^	Intensity of yellow colour within crumb/dough (pale to dark)
Aroma	Cooked milk ^1^	Heated pasteurised semi-skimmed milk
	Powdered milk (wet) ^1^	Skimmed milk powder (10% *w*/*v*, skimmed milk powder in deionised water)
	Whey isolate ^1^	Volactive Ultra-Whey 90 Instant (5% *w*/*v*, WPI powder in deionised water)
	Overall aroma intensity ^3,4^	Intensity of aroma within cupcake/scone
	Sweet ^3,4^	Sucrose (5.76 g/L)
	Lemon ^3^	Lemon zest (grated)
	Buttery ^3,4^	Cooked butter (melted unsalted butter)
	Eggy ^3^	Intensity of eggy notes
	Floury ^4^	Intensity of floury notes (self-raising flour)
	Savoury/Cheesey ^4^	Toasted cheddar cheese
	Off-Flavours ^3,4^	Curded buttermilk (cooked buttermilk)
Flavour	Sour ^1,2^	Citric acid (0.76 g/L)
	Metallic ^1,2,3,4^	Iron (II) sulphate heptahydrate (0.0036 g/L)
	Salty ^1,2^	Sodium chloride (1.19 g/L)
	Sweet ^1,2,3,4^	Sucrose (5.76 g/L)
	Cooked butter ^1,2^	Melted unsalted butter
	Cooked milk ^1,2^	Heated pasteurised semi-skimmed milk
	Powdered milk (wet) ^1,2^	Skimmed milk powder (10% *w*/*v*, skimmed milk powder in deionised water)
	Whey isolate ^1,2^	Volactive Ultra-Whey 90 Instant (5% *w*/*v*, WPI powder in deionised water)
	Overall flavour intensity ^3,4^	Intensity of flavour within cake
	Lemony ^3^	Lemon zest (grated)
	Buttery ^3,4^	Cooked butter (melted unsalted butter)
	Floury ^4^	Intensity of floury notes (self-raising flour)
	Savoury/Cheesey ^4^	Toasted cheddar cheese
	Eggy ^3^	Intensity of eggy note
	Liquorice ^3^	Liquorice (liquorice twists)
	Off-flavours ^3,4^	Curded buttermilk (cooked buttermilk)
Mouthfeel	Body ^1,2^	Fullness of sample (low to high)
	Powdery ^1,2^	Dry fine insoluble powder
	Mouthdrying ^1.2,3,4,5^	Drying sensation in the mouth
	Firmness of bite ^3,4,5^	Degree of force with first bite (soft to firm)
	Moist sponge/dough ^3,4,5^	Slightly damp sponge/dough (dry to moist)
	Chewy ^3,4,5^	Ease of ability to chew
	Greasy lips ^3,4^	Degree of oiliness/greasiness on lips
	Crumbliness of sponge/dough ^3,4,5^	Ease to break into small pieces
	Crumb size ^3^	Size of crumb inside of cake
	Pasty (cohesive) ^3,4,5^	Sticking to surfaces
	Rate of breakdown & clearance ^3,4,5^	Clearing sample from mouth (slow to fast)
	Cooling sensation ^3^	A stimulation resulting in feeling of coolness
Aftertaste	Aftertaste strength ^1,2^	The strength of the overall aftertaste
	Mouthdrying ^1,2,3,4^	Drying sensation in the mouth
	Metallic ^1,2,3,4^	Iron (II) sulphate heptahydrate (0.0036 g/L)
	Sweet ^1,3,4^	Sucrose (5.76 g/L)
	Lemon ^3^	Lemon zest (grated)
	Buttery ^3,4^	Cooked butter (melted unsalted butter)
	Savoury/Cheesey ^4^	Toasted cheddar cheese
	Off-flavours ^3,4^	Curded buttermilk (cooked buttermilk)
	Salty ^3,4^	Sodium chloride (1.19 g/L)
	Salivating ^3,4^	Increased saliva within mouth
	Liquorice ^3^	Liquorice (liquorice twists)

All anchors not to very unless otherwise stated.

**Table 4:** Overview of sensory profile modifications for each experiment.

Experiment	Panellists ^a^	Attributes ^b^	Consumption Instructions	Additional Comments
WPBs with lactose ^1^	10	18	○ Panellists assessed aroma, then consumed a sip to evaluate flavour followed by two further sips for mouthfeel and aftertaste	○ Panellists were provided with 10 mL of beverage in 25 mL plastic cups (opaque & black (BB Plastics, UK)) ○ To prevent bias evaluation, modality appearance was not assessed in case of potential visual differences
		
WPBs with fat ^2^	11	14	○ Panellists consumed a sip to evaluate taste/flavour followed by two further sips for mouthfeel and aftertaste	○ Panellists were provided with 10 mL of beverage in 25 mL plastic cups ○ To prevent bias evaluation, modality appearance was not assessed in case of potential visual differences ○ All evaluation was carried out using nose clips; therefore, aroma was also not evaluated
		
Cupcakes ^3^	10	37	○ Panellists were asked to break each cupcake in half and consume from the middle ○ Panellists assessed appearance and aroma then consumed a bite to evaluate flavour followed by two further bites for mouthfeel and aftertaste	○ Panellists were provided with a 35 g cupcake ○ All modalities were evaluated
		
Scones ^4^	10	32	○ Panellists were asked to break each scone in half and consume from the middle ○ Panellists assessed appearance and aroma then consumed a bite to evaluate flavour followed by two further bites for mouthfeel and aftertaste	○ Panellists were provided with a 30 g scone ○ All modalities were evaluated
		
Scones with and without cream topping ^5^	8	7	○ Panellists were asked to break each scone in half and consume from the middle	○ Panellists were provided with a 30 g protein scone with and without cream topping (8 g; clotted cream) ○ Only selected mouthfeel attributes were evaluated based on full sensory profiling results ^4^
		

Subscript numbers ^1–5^ reflect experiment number. ^a^ refers to the differing number of panellists present in each experiment. ^b^ denotes the varying number of attributes identified within each experiment as fully defined in Table 3. In experiments ^(1–4)^ there was a 60 s delay before scoring aftertaste and warm filtered water (~40 °C) was used as the palate cleanser in all experiments.

**Table 5:** Influence of lactose content on the sensory profile of whey protein liquid models (WPB: whey protein beverage; SF-WPB: sugar-free whey protein beverage).

Modality	Attribute	Controls			SF-WPBs Varying in Added Lactose					Significance of Sample (*p* Value)
		WPB (0.4%)	SF-WPB (0.05%)		SF-WPB (0.4%)	SF-WPB (3.4%)	SF-WPB (6.4%)	SF-WPB (9.4%)	SF-WPB (12.4%)	
Aroma	Cooked milk	12.3 ± 2.8	16.5 ± 3.9		12.9 ± 3.5	10.4 ± 2.6	16.2 ± 3.4	16.8 ± 3.6	18.4 ± 3.5	0.48
	Powdered milk (wet)	21.0 ± 3.8	18.8 ± 4.1		21.6 ± 4.1	24.9 ± 4.1	18.7 ± 4.3	22.0 ± 4.1	18.6 ± 4.0	0.72
	Whey isolate	18.6 ± 3.7	12.9 ± 3.6		13.2 ± 3.6	15.4 ± 3.1	17.6 ± 3.4	14.0 ± 2.3	13.6 ± 3.1	0.57
Flavour	Sour	22.1 ± 3.5 ^a^	24.4 ± 4.0 ^a^		21.8 ± 4.1 ^ab^	22.0 ± 3.8 ^a^	18.5 ± 3.3 ^abc^	13.7 ± 3.2 ^c^	13.8 ± 3.3 ^bc^	**0.0002**
	Metallic	12.4 ± 3.1	11.8 ± 2.8		11.6 ± 3.2	12.1 ± 2.7	8.8 ± 2.4	9.5 ± 2.1	9.1 ± 2.4	0.27
	Salty	9.5 ± 1.4	7.2 ± 2.0		7.7 ± 1.9	9.1 ± 1.8	10.2 ± 1.2	10.3 ± 1.3	7.7 ± 1.2	0.50
	Sweet	8.1 ± 2.1 ^c^	5.3 ± 1.3 ^c^		5.7 ± 2.2 ^c^	15.0 ± 2.5 ^c^	29.7 ± 3.3 ^b^	42.0 ± 1.9 ^a^	47.2 ± 2.0 ^a^	**<0.0001**
	Cooked butter	6.2 ± 1.7	2.7 ±1.4		6.3 ± 2.1	2.6 ± 1.2	4.3 ± 1.4	5.5 ± 1.6	6.4 ± 1.5	0.47
	Cooked milk	7.5 ± 2.2 ^b^	9.6 ± 2.8 ^b^		9.0 ± 2.8 ^b^	10.4 ± 2.6 ^b^	19.7 ± 3.3 ^ab^	24.5 ± 3.0 ^a^	23.8 ± 3.6 ^a^	**<0.0001**
	Powdered milk (wet)	22.3 ± 4.1	17.9 ± 4.3		23.3 ± 3.8	22.0 ± 3.9	16.6 ± 4.6	21.1 ± 4.2	19.0 ± 4.3	0.67
	Whey isolate	27.9 ± 3.1 ^a^	28.5 ± 3.9 ^a^		22.2 ± 4.0 ^ab^	25.0 ± 3.1 ^ab^	21.8 ± 3.0 ^ab^	17.7 ± 2.5 ^ab^	15.3 ± 2.6 ^b^	**0.003**
Mouthfeel	Body	30.4 ± 2.1	29.4 ± 1.9		31.6 ± 2.3	29.7 ± 2.1	26.8 ± 1.9	30.9 ± 1.9	28.4 ± 1.8	0.36
	Powdery	14.3 ± 4.2 ^ab^	11.5 ± 4.0 ^ab^		16.2 ± 4.8 ^a^	12.5 ± 3.9 ^ab^	7.5 ± 2.5 ^b^	8.6 ± 3.4 ^ab^	8.1 ± 3.1 ^ab^	**0.02**
	Mouthdrying	47.2 ± 3.6 ^ab^	49.1 ± 3.7 ^a^		45.8 ± 4.0 ^ab^	47.0 ± 3.9 ^ab^	41.7 ± 3.4 ^ab^	39.9 ± 3.1 ^b^	41.2 ± 3.1 ^ab^	**0.02**
Aftertaste	Aftertaste strength	26.9 ± 2.3 ^ab^	22.2 ± 1.8 ^b^		23.0 ± 1.9 ^b^	24.0 ± 1.9 ^b^	27.8 ± 1.5 ^ab^	30.2 ± 1.5 ^a^	27.9 ± 1.7 ^ab^	**0.0004**
	Mouthdrying	43.0 ± 3.2	45.9 ± 4.0		46.5 ± 3.6	44.2 ± 2.6	38.6 ± 3.4	41.5 ± 2.8	41.7 ± 2.7	0.32
	Metallic	7.9 ± 2.7 ^a^	6.4 ± 2.1 ^a^		8.0 ± 2.9 ^a^	5.0 ± 1.8 ^a^	3.7 ± 1.5 ^a^	3.0 ± 1.3 ^a^	4.1 ± 1.8 ^a^	**0.01**
	Sweet	4.7 ± 1.3 ^c^	3.3 ± 1.5 ^c^		5.3 ± 2.1 ^c^	7.0 ± 1.9 ^c^	18.6 ± 2.3 ^b^	27.1 ± 2.0 ^a^	30.3 ± 2.4 ^a^	**<0.0001**

Data represents means of two replicates ± standard error from trained sensory panel (*n* = 10) measured on visual analogue scales (VAS; 0–100). Differing small letters represent sample significance from multiple comparisons and brackets after sample name denote specific lactose content expressed as % *w*/*v*. All attributes are fully defined in Table 3.

**Table 6:** Influence of processing differences in whey protein powders on the sensory profile of whey protein solid models (WPC: whey protein concentrate; HS-WPC: heat-stable whey protein concentrate).

Modality	Attribute	Cupcakes			Significance of Sample (*p* Value)
		Control	WPC	HS-WPC	
Appearance	Moist appearance	52.3 ± 3.1 ^a^	26.7 ± 2.6 ^b^	19.1 ± 2.3 ^b^	**<0.0001**
	Dense appearance of sponge	39.9 ± 2.7 ^b^	56.7 ± 3.2 ^a^	64.0 ± 3.3 ^a^	**0.0001**
	Appearance of large holes in sponge	19.8 ± 2.1 ^b^	39.8 ± 4.0 ^a^	48.1 ± 4.0 ^a^	**<0.0001**
	Yellow colour of crumb (inside)	52.6 ± 1.9 ^a^	35.9 ± 2.2 ^c^	46.8 ± 2.8 ^b^	**<0.0001**
Aroma	Overall aroma intensity	53.6 ± 2.1	51.3 ± 2.4	52.2 ± 1.7	0.73
	Sweet	38.2 ± 2.9	38.4 ± 1.8	38.3 ± 1.7	1.00
	Lemon	36.7 ± 3.1	37.7 ± 3.3	37.4 ± 3.2	0.97
	Buttery	22.2 ± 3.2 ^a^	12.6 ± 2.6 ^b^	14.3 ± 2.9 ^b^	**0.003**
	Eggy	14.7 ± 2.8	13.9 ± 2.9	14.2 ± 3.1	0.98
	Off-flavours	0.0 ± 0.03	3.2 ± 1.5	4.1 ± 2.1	0.22
Flavour	Overall flavour intensity	51.6 ± 2.2	44.3 ± 2.3	48.5 ± 2.2	0.07
	Sweet	44.2 ± 3.2	38.2 ± 2.1	43.0 ± 1.9	0.22
	Metallic	0.6 ± 0.5 ^b^	4.1 ± 1.8 ^ab^	6.7 ± 2.2 ^a^	**0.04**
	Lemony	37.9 ± 2.5	32.1 ± 2.7	32.1 ± 2.3	0.28
	Buttery	23.0 ± 2.8 ^a^	8.7 ± 2.1 ^b^	11.1 ± 2.7 ^b^	**0.0005**
	Eggy	12.3 ± 2.5	9.2 ± 2.6	12.3 ± 2.8	0.55
	Liquorice	1.4 ± 1.1	5.3 ± 1.9	5.6 ± 2.7	0.23
	Off-flavours	0.0 ± 0.03	2.3 ± 1.3	3.1 ± 1.5	0.19
Mouthfeel	Firmness of bite	31.3 ± 1.7 ^b^	60.1 ± 2.5 ^a^	63.2 ± 2.8 ^a^	**<0.0001**
	Moist sponge	60.8 ± 2.2 ^a^	18.9 ± 1.6 ^b^	19.0 ± 2.6 ^b^	**<0.0001**
	Chewy	27.5 ± 2.3 ^b^	48.8 ± 4.4 ^a^	56.4 ± 3.0 ^a^	**<0.0001**
	Mouthdrying	24.5 ± 2.6 ^b^	42.3 ± 3.6 ^a^	46.3 ± 3.3 ^a^	**<0.0001**
	Greasy lips	13.7 ± 2.5 ^a^	2.3 ± 1.1 ^b^	3.2 ± 1.5 ^b^	**0.0003**
	Crumbliness of sponge	36.5 ± 3.4	33.3 ± 3.8	32.0 ± 4.1	0.76
	Crumb size	35.0 ± 2.2	45.4 ± 3.6	43.7 ± 3.9	0.06
	Pasty (cohesive)	40.0 ± 4.1	36.5 ± 3.7	36.4 ± 4.6	0.84
	Rate of breakdown & clearance	52.6 ± 3.5 ^a^	32.8 ± 1.7 ^b^	35.1 ± 2.9 ^b^	**0.0001**
	Cooling sensation	4.9 ± 2.2	3.7 ± ± 1.9	7.1 ± 2.3	0.33
Aftertaste	Mouthdrying	27.4 ± 2.7 ^b^	38.8 ± 3.5 ^a^	40.6 ± 3.8 ^a^	**0.0001**
	Sweet	39.3 ± 3.1	35.9 ± 2.8	36.8 ± 2.7	0.45
	Lemon	27.3 ± 2.8	24.5 ± 2.7	25.1 ± 2.3	0.54
	Buttery	11.3 ± 2.2 ^a^	4.9 ± 1.9 ^b^	8.6 ± 2.1 ^ab^	**0.01**
	Off-flavours	0.0 ± 0.02	1.8 ± 1.2	1.7 ± 1.0	0.36
	Salty	2.1 ± 0.9	5.8 ± 1.7	3.8 ± 1.6	0.18
	Salivating	29.4 ± 2.5	32.3 ± 3.5	34.4 ± 3.1	0.26
	Metallic	2.5 ± 1.4	6.5 ± 2.4	8.4 ± 2.2	0.06
	Liquorice	1.7 ± 1.2	2.6 ± 1.4	5.8 ± 2.6	0.11

Data represents means of two replicates ± standard error from trained sensory panel (*n* = 10) measured on visual analogue scales (VAS; 0–100). Differing small letters represent sample significance from multiple comparisons and all attributes are fully defined in Table 3.

## Data Availability

The data presented in this paper is available on request from the corresponding author. The data will be available from the University of Reading Research Data Archive upon completion of V.N. Ph.D. thesis.

## Supplementary Materials

The following are available online at https://www.mdpi.com/article/10.3390/foods10092066/s1, Table S1: Apparent viscosity (mPas; at shear rate 50 s^−1^) of whey protein liquid models. Table S2: Sensory profile of scones. Figure S1: Summary of cupcakes physical properties. Figure S2: Summary of scones physical properties.
