# Supplementary material for: Investigating Methods to Mitigate Whey Protein Derived Mouthdrying
Source: Foods. 2021 Sep 1;10(9):2066. doi: 10.3390/foods10092066 (PMC8467667; doi:10.3390/foods10092066)
Supplement: Supplementary file 1 [file foods-10-02066-s001.zip › foods-1330649-supplementary.pdf]

**Supplementary Materials:** The following are available online at www.mdpi.com/article/10.3390/foods10092066/s1, Table S1: Apparent viscosity (mPAS; at shear rate 50 $s^{-1}$) of whey protein liquid models. Table S2: Sensory profile of scones. Figure S1: Summary of cupcakes physical properties. Figure S2: Summary of scones physical properties.

Table S1 summaries the viscosity of WPBs with varying amounts of lactose and fat. The apparent viscosity of the WPB was measured following Norton et al. [7] methodology. An oscillatory rheometer (MCR 302, Anton Paar Ltd., St Albans, UK) was used and parallel plate geometry (50 mm diameter) was employed. The gap size was 1.0 mm. All samples were allowed to rest for 5-min before the measurement. Apparent viscosity was measured as a function of shear rate over the 0.001 to 1000 s-1 range at 22 °C.

**Table S1.** Apparent viscosity (mPas; at shear rate 50 $s^{-1}$) of whey protein liquid models.

| Subset | Description | Beverage Type | Apparent Viscosity |
|---|---|---|---|
| Lactose Subset | Controls | WPB (0.4%) | 1.7 ± 0.04 |
| | | SF-WPB (0.05%) | 1.7 ± 0.08 |
| | SF-WPBs (10.0%) varying in lactose levels | SF-WPB (0.4%) | 1.8 ± 0.08 |
| | | SF-WPB (3.4%) | 1.9 ± 0.2 |
| | | SF-WPB (6.4%) | 2.1 ± 0.2 |
| | | SF-WPB (9.4%) | 2.3 ± 0.02 |
| | | SF-WPB (12.4%) | 2.4 ± 0.07 |
| Fat Subset | Control | SF-WPB (0.9%) | 7.6 ± 0.04 |
| | SF-WPBs (10.0%) varying in fat levels | SF-WPB (1.8%) | 7.7 ± 0.5 |
| | | SF-WPB (3.6%) | 7.4 ± 0.8 |
| | | SF-WPB (7.2%) | 7.6 ± 1.0 |

Data represents means of six replicates ± standard error. Brackets after each sample name denote specific lactose or fat content expressed as % *w*/*v*. The grey shading demonstrates the control beverage for lactose and fat subset respectively.

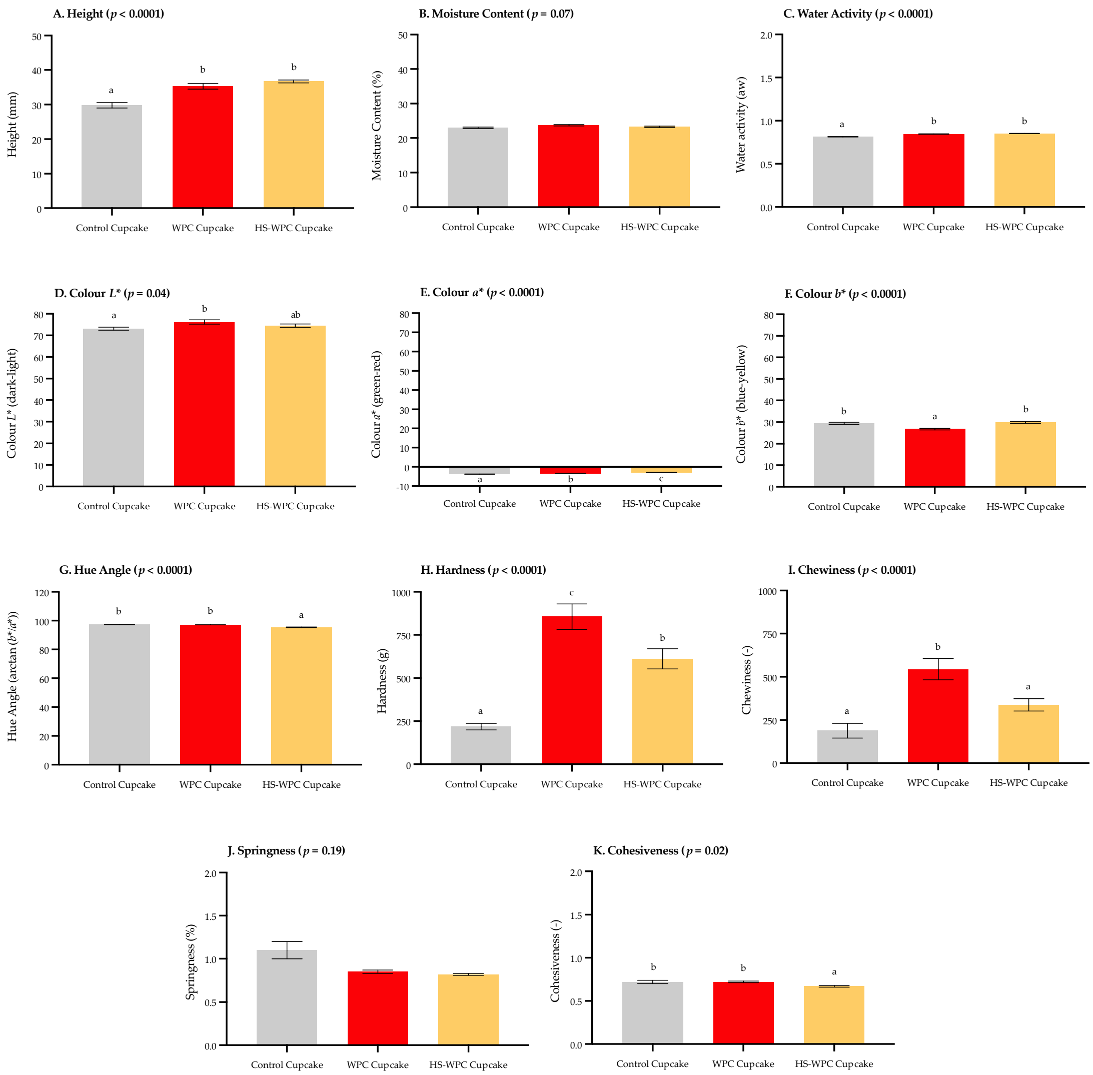


**Figure S1.** Summary of cupcakes physical properties. (WPC: whey protein concentrate; HS-WPC: heat-stable whey protein concentrate). Data represents means of three replicates from three different batches ($n$ = 9) ± standard error. Differing small letters represent sample significance from multiple comparisons and (-) denotes unitless data.

**Table S2.** Sensory profile of scones.

| **Modality** | **Attribute** | **Scones** | | **Significance of Sample ($p$ value)** |
|---|---|---|---|---|
| | | Control | Protein | |
| Appearance | Moist appearance | 34.8 ± 4.7 | 22.9 ± 3.6 | **0.005** |
| | Dense appearance of dough | 43.1 ± 3.9 | 51.5 ± 4.0 | 0.16 |
| | Appearance of large holes in dough | 25.8 ± 3.6 | 20.6 ± 2.6 | 0.15 |
| | Yellow colour of dough (inside) | 36.2 ± 3.5 | 34.4 ± 4.3 | 0.55 |
| Aroma | Overall aroma intensity | 50.1 ± 2.4 | 50.7 ± 3.9 | 0.89 |
| | Sweet | 29.3 ± 3.4 | 28.6 ± 3.1 | 0.77 |
| | Buttery | 23.2 ± 2.0 | 18.9 ± 2.5 | 0.23 |
| | Floury | 18.4 ±3.1 | 22.3 ± 3.1 | 0.12 |
| | Savoury/Cheesey | 3.4 ± 1.2 | 9.1 ± 2.5 | **0.04** |

| | | | | |
|---|---|---|---|---|
| | Off-flavours | 1.1 ± 1.0 | 2.6 ± 1.2 | 0.50 |
| Flavour | Overall flavour intensity | 43.4 ± 2.3 | 38.0 ± 3.5 | 0.17 |
| | Sweet | 24.3 ± 2.7 | 20.4 ± 2.8 | 0.29 |
| | Metallic | 0.7 ± 0.6 | 0.4 ± 0.4 | 0.27 |
| | Buttery | 19.8 ± 1.6 | 12.1 ± 2.1 | 0.054 |
| | Floury | 20.7 ± 2.6 | 25.3 ± 2.4 | 0.12 |
| | Savoury/Cheesey | 2.4 ± 1.0 | 5.5 ± 2.5 | 0.35 |
| | Off-flavours | 1.7 ± 1.2 | 0.9 ± 0.8 | 0.68 |
| Mouthfeel | Firmness of bite | 31.6 ± 2.2 | 40.3 ± 3.2 | 0.06 |
| | Moist dough | 37.3 ± 4.5 | 22.4 ± 3.1 | **0.01** |
| | Chewy | 31.0 ± 3.2 | 39.4 ± 3.4 | 0.07 |
| | Mouthdrying | 35.3 ± 3.1 | 43.9 ± 3.2 | **0.002** |
| | Crumbliness of dough | 30.1 ± 3.5 | 29.1 ± 2.5 | 0.80 |
| | Pasty (cohesive) | 35.4 ± 4.0 | 36.7 ± 4.2 | 0.69 |
| | Rate of breakdown & clearance | 40.7 ± 2.7 | 36.5 ± 3.8 | 0.34 |
| Aftertaste | Mouthdrying | 29.8 ± 2.3 | 35.8 ± 1.9 | 0.07 |
| | Sweet | 21.0 ± 2.6 | 17.9 ± 2.4 | 0.29 |
| | Buttery | 12.6 ± 1.9 | 6.7 ± 1.7 | 0.11 |
| | Savoury/Cheesey | 0.5 ± 0.4 | 1.6 ± 0.9 | 0.16 |
| | Off-flavours | 1.1 ± 0.7 | 0.4 ± 0.4 | 0.33 |
| | Salty | 2.6 ± 1.0 | 1.9 ± 0.9 | 0.56 |
| | Salivating | 23.6 ± 2.7 | 23.4 ± 3.6 | 0.96 |
| | Metallic | 2.2 ± 1.0 | 0.0 ± 0.01 | 0.11 |

Data represents means of two replicates ± standard error from trained sensory panel ($n$ = 10) measured on visual analogue scales (VAS; 0-100). All attributes are fully defined in Table 3.

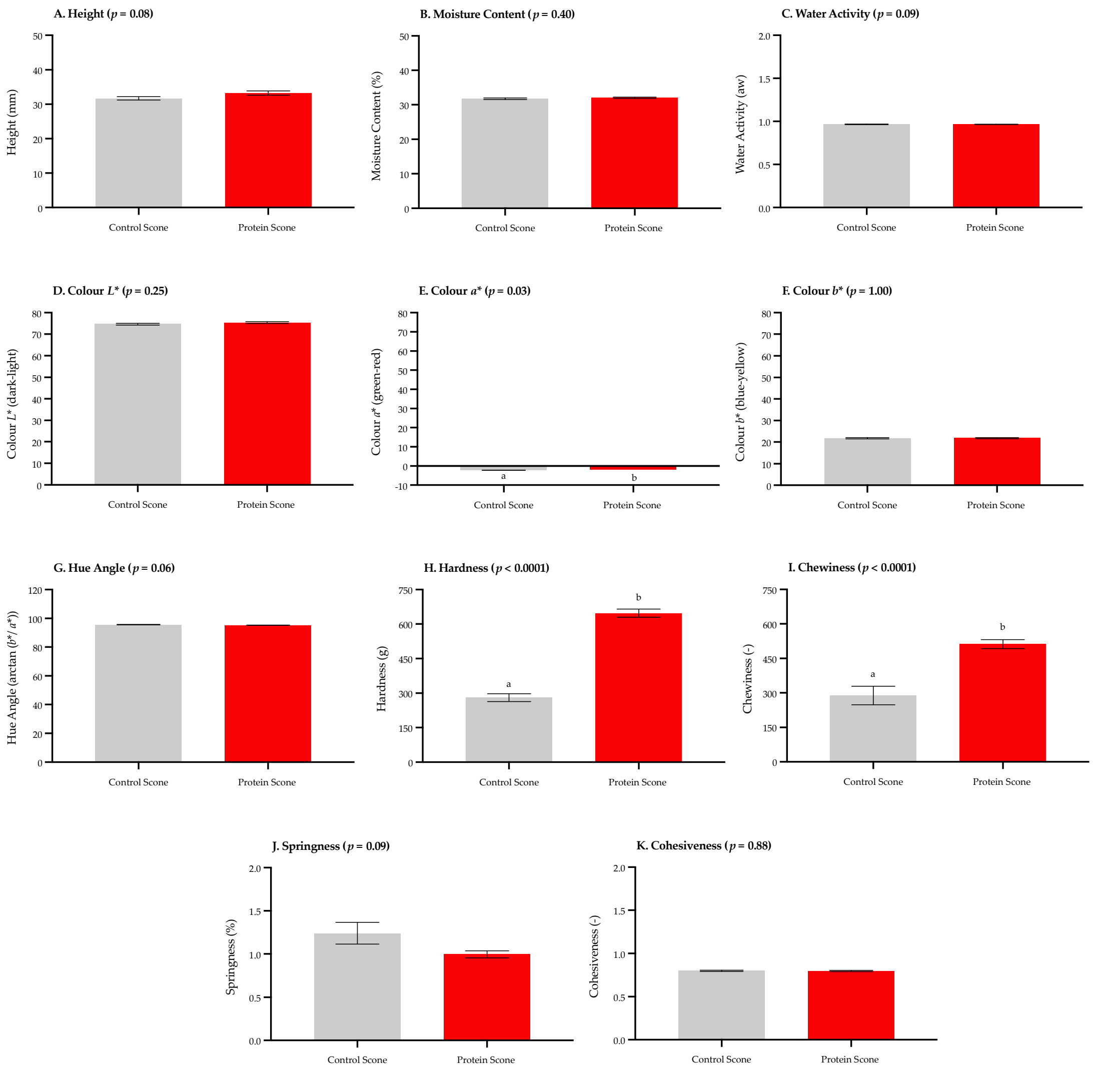


**Figure S2.** Summary of scones physical properties. Data represents means of three replicates from three different batches ($n$ = 9) ± standard error. Differing small letters represent sample significance from multiple comparisons.
